# Detoxification mechanisms of electroactive microorganisms under toxicity stress: A review

**DOI:** 10.3389/fmicb.2022.1084530

**Published:** 2022-11-29

**Authors:** Huajun Feng, Liyang Xu, Ruya Chen, Xiangjuan Ma, Hua Qiao, Nannan Zhao, Yangcheng Ding, Di Wu

**Affiliations:** ^1^School of Environmental Science and Engineering, Zhejiang Gongshang University, Hangzhou, Zhejiang, China; ^2^International Science and Technology Cooperation Platform for Low-Carbon Recycling of Waste and Green Development, Zhejiang Gongshang University, Hangzhou, Zhejiang, China; ^3^Department of Applied Chemistry, College of Chemistry and Chemical Engineering, Chongqing University of Science and Technology, Chongqing, China; ^4^Faculty of Bioengineering, Ghent University, Ghent, Belgium

**Keywords:** electroactive microorganism, toxic pollutants, toxicogenic mechanism, detoxification mechanism, extracellular electron transfer

## Abstract

Remediation of environmental toxic pollutants has attracted extensive attention in recent years. Microbial bioremediation has been an important technology for removing toxic pollutants. However, microbial activity is also susceptible to toxicity stress in the process of intracellular detoxification, which significantly reduces microbial activity. Electroactive microorganisms (EAMs) can detoxify toxic pollutants extracellularly to a certain extent, which is related to their unique extracellular electron transfer (EET) function. In this review, the extracellular and intracellular aspects of the EAMs’ detoxification mechanisms are explored separately. Additionally, various strategies for enhancing the effect of extracellular detoxification are discussed. Finally, future research directions are proposed based on the bottlenecks encountered in the current studies. This review can contribute to the development of toxic pollutants remediation technologies based on EAMs, and provide theoretical and technical support for future practical engineering applications.

## Graphical Abstract

The toxicogenic mechanisms of toxic pollutants and the detoxification mechanisms of electroactive microorganisms. 
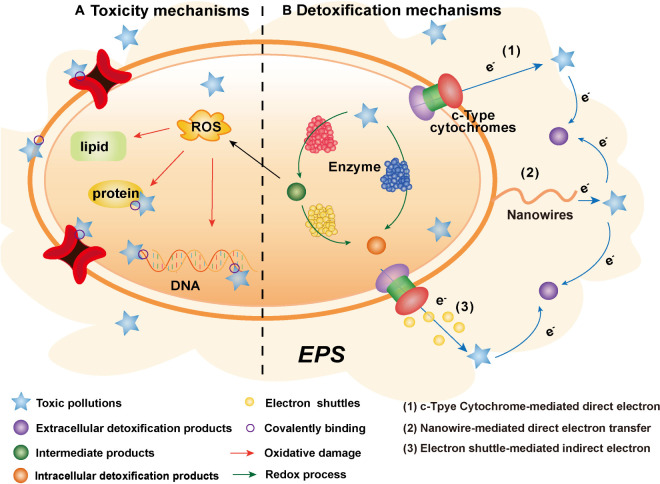

## Introduction

Toxic pollutants can be enriched in living organisms through a series of migration and transformation, resulting in carcinogenic, teratogenic, and mutagenic effect. The sources of toxic pollutants are diverse and ubiquitous in the environment (Ma et al., 2016; Zhang et al., 2016; Sun et al., 2022b). Treating toxic pollutants efficiently has always been a focus and a difficult task in the field of environmental remediation.

Microbial remediation is the most commonly used technology to treat toxic pollutants, but toxicity stress causes a bottleneck in the efficient and stable microbial remediation. Microorganisms generally transfer toxic pollutants to cells for metabolism, achieving detoxification by degrading the pollutants. However, degradation and detoxification are sometimes not completely synchronous. Liu et al. (1998) found that although pyridine was degraded by microorganisms, the microbial toxicity increased with the formation of the intermediate methylpyridine in this process, thus inhibiting the activity of microorganisms and even leading to system collapse.

It has been reported that electroactive microorganisms (EAMs) show better activity than that of other microorganisms under toxicity stress (Zheng et al., 2020; Chaudhary et al., 2022). *Desulfovibrio*, an electroactive sulfate-reducing bacteria, became the dominating bacteria at the genus level and accounted for 32% of the relative abundance after 150 days under the toxicity stress of Cr(VI), despite the fact that it was scarce in the initial inoculum (Qian et al., 2022). The most likely explanation for this phenomenon is that EAMs have a unique metabolic pathway that can perform extracellular electron transfer (EET) with redox active substances to complete a series of metabolic activities (Li and Yu, 2015; Logan et al., 2019). However, the correspondence between the extracellular metabolism and the detoxification ability of EAMs and the related mechanisms still need to be clarified further.

The toxic pollutants remediation technology based on EAMs has the advantages of environmental friendliness and low cost, considered to be the most green and sustainable new environmental pollution treatment method at present (Li and Yu, 2015; Xu et al., 2019). Despite various reviews on the EET mechanism of EAMs (Xiao and Yu, 2020; Xie et al., 2020; Thapa et al., 2022), there haven’t many relevant summaries that are pertinent to the detoxification mechanism of EAMs under pollutants’ toxicity stress. This review summarizes the toxicogenic mechanisms of toxic pollutants to microorganisms, the detoxification mechanisms of EAMs to these pollutants and several strategies to strengthen extracellular detoxification, as well as prospects for possible future research directions based on conclusions regarding the problems encountered in current research.

## Toxicogenic mechanisms of toxic pollutants to microorganisms

**FIGURE 1:**
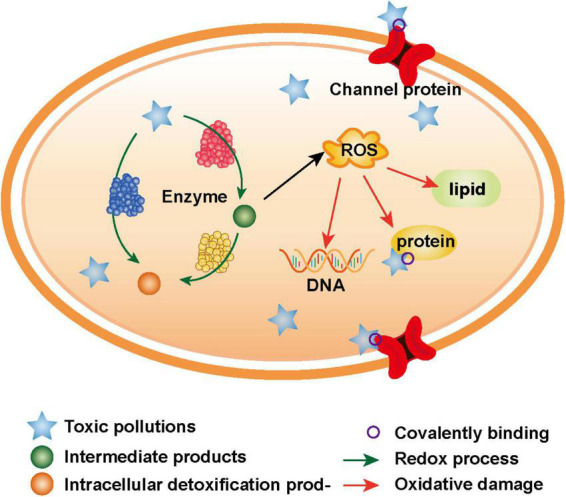
Toxicogenic mechanisms of toxic pollutants.

There are two main toxicogenic mechanisms of toxic pollutants to microorganisms, including the following: on the one hand, the electrophilic groups of toxic pollutants can directly covalently bond with the nucleophilic groups of biological macromolecules (Dudev and Lim, 2014); on the other hand, numerous reactive oxygen species (ROS), which are produced in the oxidation-reduction process of toxic pollutants, oxidize biological macromolecules (Figure 1; Guo et al., 2021). The essence of these two mechanisms is that toxic pollutants with strong electrophilicity directly or indirectly react with biological macromolecules to destroy the structure and function of cells, finally leading to apoptosis (Roy et al., 2005).

### Covalent binding to biological macromolecules

In microbial cells, the electrophilic groups of toxic pollutants or their metabolites can covalently bind with nucleophilic groups in biological macromolecules, thus changing the structure and function of biological macromolecules, such as DNA and proteins, and causing a series of harmful biological effects (Jiang et al., 2022).

Because its bases contain many nucleophilic sites, DNA can covalently bind with electrophilic toxic pollutants or their metabolites to form stable complexes (Shibata and Uchida, 2019). For example, heavy metals can covalently bind with the nitrogen atoms located on DNA bases (Tanaka and Ono, 2008); the intermediate product (high-activity electrophilic diol epoxide), which is produced in the intracellular metabolic process of polycyclic aromatic hydrocarbons in microbes, can covalently bind with deoxyguanosine (Vondracek and Machala, 2021). There are two ways to cross-link DNA by toxic pollutants, including intrachain interactions and interchain interactions, and the formation of complexes causes DNA double strands to unwind and bend (Park et al., 2005).

Because of its nucleophilic groups (e.g., amino, sulfhydryl, and carboxyl), proteins can also be covalently modified, resulting in various harmful biological effects (Wells and Winn, 1996). For example, acetaldehyde can covalently bind with various proteins (e.g., albumin, erythrocyte protein, tubulin, lipoprotein, and cytochrome enzymes involved in ethanol metabolism) to form complexes, thus interfering with the physiological functions of proteins and stimulating the immune response of cells (Israel et al., 1986; Smith et al., 1989; Wehr et al., 1993). Moreover, Mauch et al. (1986) proved that the free lysine e-amino group is an important target of proteins that covalently bind with acetaldehyde. In addition, heavy metals can bind with thiol groups that are located on proteins and damage protein folding or the combination of cofactors and enzymes, thus destroying the normal biological activity of proteins (Sharma and Melkania, 2018).

### Accumulation of reactive oxygen species

Reactive oxygen species are oxygen derivatives, such as superoxide anions, hydroxyl radicals, and hydrogen peroxide, which are produced by enzymatic reactions during the normal metabolic activities of microorganisms (Sun et al., 2022a). Under normal physiological conditions, the content of intracellular ROS in microorganisms is dynamically balanced, and a small amount of ROS can be removed by the antioxidant system. However, due to the limited scavenging capacity of antioxidant systems, excessive ROS may damage the activities and functions of various antioxidant-related enzymes (Ballard and Towarnicki, 2020; Sies and Jones, 2020; Sun et al., 2022a). The intake of toxic pollutants (e.g., polycyclic aromatic hydrocarbons and heavy metal ions) can promote the production of ROS (Gao et al., 2019; Vondracek and Machala, 2021). Excessive ROS mainly act on lipids, proteins and DNA in cells, causing oxidative damage and finally affecting the normal physiological and metabolic activities of microorganisms (Tekpli et al., 2011).

When a large amount of ROS accumulates in cells, the rich lipids in the cell membrane are greatly damaged. The peroxidation of lipids may change the physiological characteristics of the membrane and disturb the lipid asymmetry of the membrane, thus changing the fluidity of the membrane. As a result, membrane depolarization and an eventual loss of integrity occurs (Tekpli et al., 2011). With a microbial electron microscope, Morcillo et al. (2016) observed that the structure of the microbiological cell membrane was obviously damaged after exposure to toxic heavy metals. However, damage to the membrane structure not only affects the process of microbiological substrate intake but also promotes the accumulation of toxic pollutants in cells, thus aggravating the toxicity effects on microorganisms (Chen et al., 2020b).

Reactive oxygen species undergo oxidation reactions with intracellular structural and functional proteins, and these reactions include the removal of carboxyl groups, formation of carbonyl groups, and oxidation of sulfhydryl groups (Wells et al., 1997). For example, iron will react with hydrogen peroxide to generate more active ⋅OH (Fenton reaction), leading to the oxidation of amino acids such as histidine, tyrosine, L-cysteine (Yan et al., 2021). The oxidative damage caused by these reactions leads to the crosslinking of proteins, changes in amino acid composition and protein structure, which will change or destroy the function of the protein (Davies et al., 1991).

Reactive oxygen species can also directly destroy the double-stranded structure of DNA through the shear action of oxidation reactions (Radzig et al., 2013). Thiebault et al. (2007) found that a high concentration of U could induce the production of ROS and cause irreversible damage to DNA. Not all ROS cause oxidative damage in the same way, ⋅OH can directly react with DNA, while other less active ROS is through a series of reactions into a higher active ROS such as ⋅OH and ⋅ONOO^−^ playing an oxidizing role. For example, $\cdot {\text{O}}_{2}^{-}$ can’t directly lead to DNA damage, but can react with NO to generate ONOOH, resulting in oxidative damage to DNA (van der Vliet et al., 1995).

## Detoxification mechanisms of electroactive microorganisms

Since contact with the cell membrane and entry into cells is a prerequisite for the pollutants’ toxicity effects on EAMs, microorganisms generally produce a series of responses in the cell to achieve detoxification under toxicity stress. However, due to the limited capacity of this intracellular detoxification, it is difficult for microorganisms to maintain good activity for a long time under toxic stress. Recent studies have reported that EAMs perform better activity than normal microorganisms under toxicity stress (Zheng et al., 2020; Chaudhary et al., 2022). Unlike normal microorganisms, EAMs can transfer electrons to the extracellular space through their unique EET pathway (Chaudhary et al., 2022). At the same time, toxic pollutants with strong electrophilicity can be used as the final electron acceptors in the process of extracellular respiration due to their good ability to accept electrons (Roy et al., 2005). Therefore, in addition to intracellular detoxification, EAMs can also detoxify toxic pollutants through the EET pathway.

### Intracellular detoxification

Under suitable environmental conditions, EAMs can detoxify toxic pollutants by their intracellular metabolic activity, similar to other microorganisms. The intracellular metabolic activity of EAMs also depends on the intracellular electron transfer (IET) pathway, which is mainly composed of three parts, namely, various functional enzymes, quinone pools (e.g., menadione or ubiquinone) and c-type cytochromes (cyt-cs) (Chen and Strous, 2013). Among them, quinone pools can mediate electron transfer between the cytoplasm and periplasm; cyt-cs can play a role in electron transfer among various enzymes (Yang et al., 2020).

Enzymes play an important role in the IET pathway, and several studies have proven that they mediate nearly all reactions in organisms (Zumstein and Helbling, 2019). In the process of detoxification of toxic pollutants by EAMs, a complete IET pathway often contains a variety of enzymes, such as monooxygenase, dioxygenase, and dehydrogenase, which may be needed in the detoxification of complex organics such as polycyclic aromatic hydrocarbons (PAHs) by EAMs (Ismail et al., 2022). The O_2_ content in the environment affects the detoxification of toxic pollutants by EAMs (Alegbeleye et al., 2017). The first step in the detoxification of PAHs by EAMs (e.g., *Pseudomonas aeruginosa* and *Ochrobactrum anthropi*) under aerobic conditions is to reduce the number of benzene rings, which occurs in two ways: through monooxygenase and dioxygenase (Logan et al., 2019; Ismail et al., 2022). Monooxygenase combines an oxygen atom into PAHs to form oxidized aromatic hydrocarbons, and then hydrates with epoxidase to form *trans*-dihydrodiol or spontaneously isomerizes to form phenols (Fuchs et al., 2011). Dioxygenase decomposes the aromatic ring to form *cis*-dihydrodiol, which is then transferred to NAD^+^ by dehydrogenase while the important intermediate product catechol is metabolized (Alegbeleye et al., 2017). Finally, the hydrolase opens the ring of catechol in the ortho position to form *cis, cis*-muconic acid or opens the ring by ring transformation to form 2-hydroxymuconic semialdehyde (Seo et al., 2009). The detoxification of PAHs by microorganisms can also occurs under anaerobic conditions through using inorganic salts, such as nitrate and sulfate, and metal ions, such as trivalent iron and high-valent manganese, as electron acceptors for respiration to oxidize PAHs to low molecular weight substances (Nzila, 2018). Similarly, EAMs can detoxify Cr(VI) intracellularly under aerobic and anaerobic conditions. Under aerobic conditions, reductants such as Cr(VI) reductase, dimer glycoprotein, and *n*-ethylmaleimide reductase can mediate the reduction reaction of Cr(VI). Under anaerobic conditions, water-soluble and fat-soluble reductase such as cytochrome enzyme, hydrogenase, and flavin reductase mediate the reduction of Cr(VI) (Thatoi et al., 2014; He et al., 2020).

Although intracellular detoxification can transform toxic pollutants into low-toxicity substances, sometimes the toxicity effects on EAMs are enhanced in the process. Formation of toxic intermediates (Table 1) and ROS is the reason for increased toxicity. In addition, due to the limited intracellular detoxification, there are still some toxic pollutions can directly cause toxicity effects on EAMs (Rathore et al., 2022). At the same time, the toxicity of these pollutions can attack enzymes as a class of proteins (Feng et al., 2015). Therefore, the intracellular detoxification effect of EAMs can be inhibited under toxic stress. Chang et al. (2016) reported that two homologous strains, Trichoderma asperellum PTN7 and PTN10, exhibited different tolerance and detoxification capacity under the toxicity stress of Cr(VI). This is mainly related to the different reduction capacities of the two strains to extracellular crude enzyme. Due to its superior extracellular reduction ability, PTN10 can alleviate the process of DNA and protein damage caused by ROS produced during the intracellular reduction of Cr(VI), thus showing better toxicity tolerance and biological activity than PTN7 (Xia et al., 2021).

**TABLE 1:** A list of microbial toxicity of parent pollutants weaker than metabolites.

Pollutant	Intermediate/Final product	References
Pentachlorophenol	Tetra-chloro-benzoquinone and 2,5-dichloro-3,6-dihydroxy-1,4-benzoquinone	Bryant and Schultz, 1994
Phenanthrene	Pyrogallol	Wang et al., 2021
2,4-Dichlorophen oxyacetic acid	2,4-Dichlorophenol	Seck et al., 2012
Trichloroethylene	Vinyl chloride	Dolan and McCarty, 1995
Nitrosamine	Alpha-hydroxynitrosamine	Loeppky and Shi, 2008
Inorganic arsenic	Methylarsonic acid and dimethylarsinic acid	Vahter, 2002
Inorganic lead	Trimethyl lead and triethyl lead	Li X. X. et al., 2020
Inorganic mercury	Methylmercury	Pan-Hou, 2010

In conclusion, the intracellular metabolism of EAMs can detoxify toxic pollutants, but compared to extracellular detoxification, this method is more negative. Because contact with the cell membrane and entry into cells is a prerequisite for the toxicity effects of toxic pollutants on EAMs, intracellular detoxification cannot fundamentally play a detoxification role, which explains why normal microorganisms are unable to maintain long-term good activity in the environment under toxic stress.

### Extracellular detoxification

The extracellular detoxification mechanism of EAMs is mainly to reduce the electrophilicity of toxic pollutants by extracellular reduction, thereby reducing the pollutants’ toxicity effects (Chen et al., 2019; Cui et al., 2021). Nitroaromatic hydrocarbons are common toxic organic pollutants. Due to the strong electrophilicity of the nitro groups located on benzene rings, nitroaromatic hydrocarbons are not easily oxidized by microorganisms but are easily reduced (Tas and Pavlostathis, 2014). It has been reported that EAMs can achieve the extracellular reduction of nitroaromatic hydrocarbons through the EET pathway. For example, *Geobacter metallireducens* and *Shewanella putrefaciens* CN32 can transfer the electrons obtained by the oxidation of substrates to nitroaromatic hydrocarbons through extracellular iron oxides, thus promoting the reduction of nitro groups to produce amino aromatic hydrocarbons with low toxicity (Tobler et al., 2007; Luan et al., 2015). In addition, the extracellular reduction of EAMs can also act on toxic inorganic pollutants, such as Cr(VI) (Xia et al., 2021). Because Cr(VI) is reduced to Cr(III) with low electrophilicity through extracellular reduction, its toxicity to EAMs decreased to 1/100 of the original level (Chang et al., 2016). Therefore, the extracellular reduction of toxic pollutants by EAMs is an effective detoxification mechanism (Table 2).

**TABLE 2:** A summary of toxic pollutants reduced by electroactive microorganisms.

Pollutant	Electron acceptor	Electron donor	Microorganism	Reaction efficiency	Reaction rate	References
1,3-Dinitrobenzene	Nitro group	Reducing sugar	*Bacillus subtilis*	73.2% from 2.19 mg/L in 10 h	0.136 h^–1^	Zhou et al., 2020
			*Escherichia coli* DH5α	91.7% from 2.19 mg/L in 3 h	0.84 h^–1^	
Nitrobenzene	Nitro group	Sodium lactate, 10 mM	*Shewanella putrefaciens* CN32	/	1.31 d^–1^	Luan et al., 2015
				/	1.88 d^–1^	
				/	1.53 d^–1^	
Cationic red X-GRL	Azo bonds	Lactate, 18 mM	*Shewanella oneidensis* MR-1	98.4% from 100 mg/L in 12 h	0.176 h^–1^	Li et al., 2018b
Methylene blue	Cyano	NaBH_4_	*Bacillus cereus*	98% from 50 mg/L in 1 h	2.983 h^–1^	Alfryyan et al., 2022
Potassium dichromate	Cr(VI)	Lactate, 30 mM	*Shewanella oneidensis* MR-1	80% from 25 mg/L in 6 h	0.255 h^–1^	Liu et al., 2020
Potassium dichromate	Cr(VI)	Sodium acetate, 0.1 g/L	Mixed bacteria	90.8% from 1 mg/L in 144 h	/	Beretta et al., 2020
/	Cu(II)	Sodium acetate, 1 g/L	Mixed bacteria	97.8% from 5 mg/L in 72 h	/	Zhang et al., 2018b
NaVO_3_⋅2H_2_O	V(V)	Methanol phenanthrene	Mixed bacteria	100% from 10 mg/L in 168 h	0.44 d^–1^	Shi et al., 2020

**FIGURE 2:**
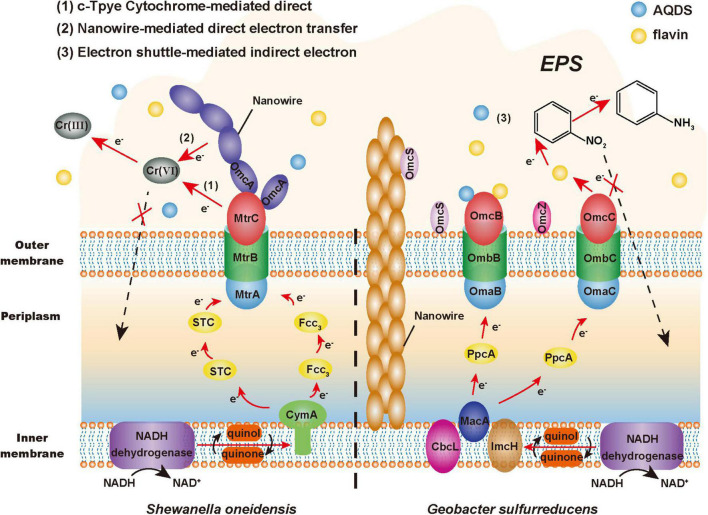
Extracellular detoxification mechanism of electroactive microorganisms (EAMs). The red arrow represents the electron transfer pathway, the black arrow represents the material transformation process, and the dashed arrow represents the non-occurring toxicity effects.

Electroactive microorganisms, as a complex with strong catalysis, promote the extracellular redox process of toxic pollutants to achieve detoxification (Xie et al., 2020). The EET pathway is the key to the extracellular detoxification ability of EAMs, which is composed of a series of redox processes (Figure 2). And the EET pathways of EAMs are mainly composed of cyt-cs, nanowires and small molecule electroactive substances (Zhao et al., 2021).

A large number of studies have found that a variety of cyt-cs with different positions and functions are involved in the EET pathway, such as CymA inner membrane protein, Fcc_3_ and STC periplasmic protein, MtrA and OmcA outer membrane protein in the model bacterium *Shewanella oneidensis* MR-1 (Zhao et al., 2021); MacA inner membrane protein, PpcA periplasmic protein, OmcS and OmcZ outer membrane protein in the model bacterium *G. sulfurreducens* (Mehta et al., 2005; Nevin et al., 2009). It has been reported that the cyt-cs of EAMs can reduce U(VI) and Cr(VI) *in vitro* (Shelobolina et al., 2007; Liu et al., 2016). This series of proteins constitute the electron transfer chain of EAMs, such as the Mtr respiratory pathway of MR-1. Wang et al. found that the reduction ability of the MR-1 mutant with the deficiency of CymA to nitroaromatic hydrocarbons was 64.3% lower than that of wild-type bacteria, which indicated that CymA played an important role in the detoxification ability of MR-1 (Wang et al., 2020). In addition, it is estimated that the periplasmic space (approximately 0.2 fL) of an MR-1-cell contains more than 300,000 hemes from cyt-cs (Sturm et al., 2015). This means that EAMs may have multiple EET pathways. For example, its similar homologous complex MtrDEF can also play a role in compensating for the electron transfer function when MtrABC is blocked (McLean et al., 2008). In general, these cyt-cs constitute a dense electron transfer network, which provides multiple EET pathways for EAMs to reduce and detoxify toxic pollutants extracellularly.

Nanowires, which are conductive appendages and filaments that can transmit electrons extracellularly, can also be used by EAMs to transfer electrons across extended distances (Gorby et al., 2006; Shi et al., 2016). *Shewanella* can generate nanowires with a length of tens of microns by extending the fusion of MtrC and OmcA to the outside of cells (El-Naggar et al., 2010; Pirbadian et al., 2014). However, the nanowires of *Geobacter* are pili composed of PilA protein, and their electrical conductivity may come from the aromatic amino acids located on PilA protein or OmcS with electrical conductivity (Vargas et al., 2013; Liu et al., 2019). According to previous research reports, nanowires exhibit a good performance of extracellular catalytic reduction under the stress of heavy metals. *G. sulfurreducens* can transfer electrons to extracellular U(VI) by conductive pili and then reduce it to insoluble U(IV), which precipitates on the surface of pili to achieve extracellular detoxification (Cologgi et al., 2011; Reguera, 2018). Therefore, this electron transfer mechanism can reduce the harm to microorganisms from toxic pollutants within the conductive range of nanowires and plays a protective role.

Due to the good redox properties, some endogenous (e.g., phenazines, flavins, and quinones) and exogenous [e.g., anthraquinone-2,6-disulfonate (AQDS), cysteine, and sulfur-containing molecules] small-molecule electroactive substances can flexibly mediate and promote the EET process (Huang et al., 2018). These substances are called electron shuttles (Wu et al., 2020). Marsili et al. (2008) found that the efficiency of electron transfer to the electrode of the MR-1 biofilm was reduced by more than 70% after removing flavins. Conversely, the addition of electron shuttles can enhance the detoxification ability of EAMs. Chen et al. found that the addition of AQDS promoted electron transfer between soil microorganisms and iron minerals, thus producing more active Fe(II) to promote the dechlorination of pentachlorophenol (Chen et al., 2020a). Therefore, electron shuttles can make a great contribution to promoting the process in which EAMs transfer electrons extracellularly to toxic pollutants by the EET pathway.

In addition, the extracellular polymeric substances (EPSs) that are tightly attached to the cell membrane surface have a certain auxiliary effect on the extracellular detoxification of EAMs. On the one hand, EPSs can be regarded as a “huge” protective barrier, in which rich functional groups, such as carboxyl, hydroxyl, and sulfonyl groups, actively participate in the binding of toxic pollutants (Maurya et al., 2022). On the other hand, EPSs are important interface mediums for communication and materials exchange between EAMs and the external environment, particularly when used as electron shuttles (e.g., polysaccharide, protein, and humic acid) can promote the EET process (Sedenho et al., 2021). Jiang and Kappler (2008) found that *G. sulfurreducens* used dissolved humic substances as electron shuttles to transfer electrons to iron oxide at a rate approximately seven times higher than that of direct transfer. Interestingly, the hydrophobicity and permeability of cell membrane will change under toxic stress, which promotes the release of macromolecules such as proteins and polysaccharides, thus increasing the content of EPSs on the surface of EAMs and improving their detoxification effect (Maurya et al., 2022).

In summary, EET plays an important role in the extracellular detoxification of toxic pollutants by EAMs. In terms of EET, the abundant cyt-cs in cells are the main source of the ability of EAMs, constituting various electron transmission pathways and playing a supporting role in the whole EET process. While the endogenous or exogenous electron shuttles and EPSs with good redox activity can also flexibly promote electron transfer (Li et al., 2016).

## Strengthening the extracellular detoxification strategies

**FIGURE 3:**
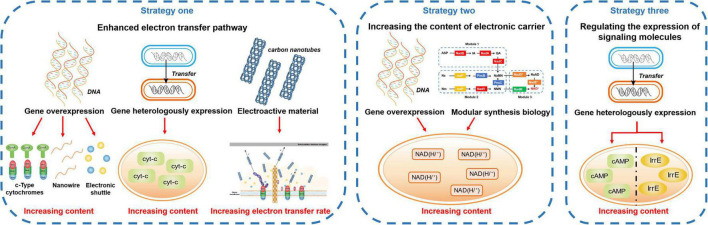
Strengthening the extracellular detoxification strategies.

Compared with extracellular detoxification, intracellular detoxification is a passive response to the stress of toxic pollutants. EAMs can detoxify toxic pollutants in cells, but the effect of toxicity on EAMs cannot be prevented. Therefore, extracellular detoxification under toxicity stress is more beneficial for EAMs to survive in an environment of toxic pollutants. However, the relatively inefficient electron transfer between electron acceptors and donors is the bottleneck that prevents EAMs from using the ability of EET to detoxify toxic pollutants (Wang et al., 2022). Therefore, if the EET ability of EAMs can be improved by some strategies, the extracellular detoxification ability of EAMs to toxic pollutants may be strengthened (Figure 3).

### Enhanced electron transfer pathway

The EET pathway of EAMs is mainly composed of cyt-cs, small-molecule electroactive substances, and nanowires. Among them, cyt-cs play a supporting role in the whole EET process (Chiranjeevi and Patil, 2020); nanowires play an important role in the long-distance EET of EAMs (Lovley, 2011); small molecule electroactive substances can be used as electron shuttles to flexibly transfer electrons (Watanabe et al., 2009). At the same time, it has been found that some nano scale artificial conductive materials can also promote the EET process of EAMs (Zhao et al., 2021). Therefore, increasing the content of cyt-cs, nanowires, small-molecule electroactive substances and artificial conductive materials may be a strategy to promote the EET process of EAMs and strengthen their detoxification ability.

Studies have proven that biosynthesis can regulate the expression of related genes to promote the synthesis of cyt-cs that are related to the EET pathway in EAMs, thus strengthening their detoxification ability (Rosenbaum and Henrich, 2014). Li Y. et al. (2020) induced the overexpression of cyt-cs (e.g., MtrA, MtrB, MtrD, and OmcA) genes of *S. xiamenensis* by graphene oxide, thereby increasing the ability to reduce Cr(VI) by 2.7 times. In addition, some researchers have promoted the EET ability of other EAMs by the heterologous expression of cyt-cs genes with good EET performance. For example, Jensen et al. (2010) reconstituted MtrA, MtrB, and MtrC on the EET pathway of *S. oneidensis* MR-1 into *Escherichia coli*, thereby making the ability to reduce metal ions and their oxides of engineered strains eight times and four times higher than that of the parent wild-type, respectively.

Increasing the content of electron shuttles in the EET pathway can also improve the detoxification ability of EAMs (Liu et al., 2018). On the one hand, add exogenous electron shuttles directly. For example, Kang et al. (2022) found that under the condition of Fe(III) reduction, the addition of sucrose and NH_4_^+^ enhanced the reduction ability of EAMs to perfluoroalkyl substances in sediments, while the system with added NH_4_^+^ had the best enhancement effect, and the remaining perfluoroalkyl substances in this system decreased by 30.2% compared with that of the control group after 5 days. On the other hand, promote the generation of endogenous electron shuttles by regulating gene expression. Yang et al. (2022) heterologously expressed the riboflavin synthesis gene cluster *ribADEHC* of *Bacillus subtilis* in *S. carassii*-D5, thus the riboflavin yield and the reduction efficiency of methyl orange were 4.7 times and 1.3 times that of the wild type, respectively.

It has been reported that promoting the synthesis of nanowires can strengthen the EET ability of EAMs. For example, Leang et al. (2013) knocked out the PilZ protein coding gene GSU1240 in the *G. sulfurreducens* genome to make engineered strains produce more nanowires, while the maximum current density increased by 1.5 times. However, at present, few studies have reported that this strategy can improve the detoxification ability of EAMs. As nanowires play an important role in the long-distance and interspecific electron transfer process of EAMs, it is speculated that this strategy may help to strengthen the detoxification ability of EAMs to extracellular toxic pollutants and promote the synergistic detoxification among EAMs.

Recent studies have found that carbon nanotubes with good biocompatibility and excellent electrical conductivity can promote the redox process of cyt-cs (Zhang et al., 2018a). To explore whether carbon nanotubes can strengthen the detoxification ability of EAMs, Yan et al. (2013) found that most nitrobenzene was reduced extracellularly by adding 0.5% (w/v) carbon nanotubes to the cell-immobilized alginate beads, and the reduction efficiency of nitrobenzene increased by 74%. In addition, it was observed that the strain showed better activity under toxicity stress. It is speculated that the reason why CNTs can change the electron transfer mechanism of EAMs is as follows: (1) CNTs are rich in redox-active sites that are conducive to high conductivity, thus accelerating EET (Tasis et al., 2006); (2) CNTs can absorb a variety of organic compounds (Chen et al., 2007; Zhang et al., 2009), thus allowing greater mass diffusion and enhancing reaction kinetics. Additionally, whether other nanomaterials with similar physical and chemical properties to carbon nanotubes have this function is worthy of further study (Guo et al., 2015; Liu et al., 2015; Huang et al., 2017).

### Increasing the content of electronic carrier

NAD (H/^+^) in EAMs is an important carrier of electrons in the EET pathway, and it is also among the key limiting factors of its detoxification ability (Li et al., 2018a). Therefore, regulating the synthesis of intracellular NAD (H/^+^) may solve the problem involving the low electron transfer efficiency in the EET pathway by promoting the generation of electrons, thus strengthening the detoxification ability of EAMs (Yong et al., 2014). Yong et al. (2014) significantly increased the availability of NAD (H/^+^) by overexpressing the NAD synthase gene *nadE*, thereby making the output power of the engineered strain 3 times higher than that of the wild-type strain. Additionally, several studies have applied the strategy of modular synthesis biology, such as Li et al. (2018a) redirected the synthesis of NAD^+^ by using three modules (*ab initio*, remediation, and general biosynthesis) of *S. oneidensis* MR-1, thereby increasing the content of NAD (H/^+^) in cells and increasing the number of electrons entering the EET pathway. Similarly, whether this strategy can strengthen the detoxification ability of EAMs needs further exploration.

### Regulating the expression of signaling molecules

Intracellular signaling molecules can influence the growth and metabolic activity of EAMs by regulating the expression of related genes, such as the second messenger or global regulator, which can regulate its EET pathway (Camilli and Bassler, 2006). Therefore, whether the detoxification ability of EAMs can be strengthened by regulating the expression of intracellular signaling molecules has attracted the attention of many researchers. Cheng et al. (2020) heterologously expressed the adenylate cyclase-encoding gene from *Beggiatoa* sp. PS in *S. oneidensis* MR-1 to increase intracellular second messenger cyclic adenosine 3’, 5’-monophosphate (cAMP) content, thereby upregulating the expression level of the coding genes of the cyt-cs and flavin synthesis pathways, resulting in the reduction rate of Cr(VI) being increased by three times higher. Additionally, several studies have found that the EET efficiency of EAMs can also be improved by regulating intracellular global regulatory factors. For example, Luo et al. (2018) introduced the global regulatory factor IrrE of *Deinococcus radiodurans* into *P. aeruginosa* PAO1, thereby promoting the secretion of more phenazine compounds and increasing the maximum power density of the system by 70%. Therefore, regulating the expression of intracellular signaling molecules is a feasible strategy to strengthen the detoxification ability of EAMs.

## Conclusion and outlook

Toxicity stress has a strong detrimental impact on microbial activity during the bioremediation of toxic pollutants. Under toxicity stress, EAMs exhibit more steady activity than ordinary microorganisms, which is mostly due to EAMs’ unique and outstanding detoxifying capabilities. However, the detoxification mechanisms of EAMs are still lack relevant comprehensive review. This review summarizes the toxicogenic pathways of toxic pollutants to microorganisms, the detoxification mechanisms of EAMs and important strategies to strengthen extracellular detoxification:

1) The main toxicogenic mechanisms of toxic pollutants include direct covalent binding of toxic pollutants’ electrophilic groups to nucleophilic groups of biological macromolecules, as well as the promotion of ROS generation causing oxidative damage to cells.
2) Under toxicity stress, the detoxification mechanisms of EAMs to toxic pollutants are included intracellular detoxification and extracellular detoxification. The excellent detoxification ability of EAMs comes from its extracellular detoxification mechanism, which can extracellularly reduce electrophilic toxic pollutants through EET pathway.
3) The extracellular detoxification capacity of EAMs can be further improved by enhancing the electron transport pathway, regulating the synthesis of NAD (H/^+^) and modulating signaling molecule expression.

However, the study on detoxification of EAMs under toxicity stress is still in the initial stage, with most studies focusing on a single pollutant and rarely on the combined effects of pollutants. Therefore, comprehensive researches into the detoxification process of EAMs on composite contaminants are required for improved applicability in practical engineering. At the same time, it is unknown whether the metabolic activities of other microorganisms coexisting with EAMs in practical applications will assist or interfere EAM detoxification. Furthermore, many strategies have been demonstrated to enhance the ability EET, but some of them have not been applied to enhance their detoxification ability under toxicity stress, thus the EET ability of EAMs and the mechanism of enhancing their detoxification ability remain to be further explored. Further clarification of these understandings may help us to develop EAMs-based toxic pollutants remediation technologies, as well as providing a more complete theoretical basis for future practical engineering applications.

## Author contributions

HF: funding acquisition, conceptualization, writing—review and editing, and supervision. LX: investigation, visualization, formal analysis, and writing—original draft. RC: conceptualization, methodology, and writing—review and editing. XM: formal analysis and writing—review and editing. HQ: formal analysis and writing—polishing and editing. NZ: investigation and writing—review and editing. YD: writing—review and editing. DW: supervision. All authors contributed to the article and approved the submitted version.
